# The impact of quality and accessibility of primary care on emergency admissions for a range of chronic ambulatory care sensitive conditions (ACSCs) in Scotland: longitudinal analysis

**DOI:** 10.1186/s12875-019-0921-z

**Published:** 2019-02-22

**Authors:** Marjon van der Pol, Damilola Olajide, Mark Dusheiko, Robert Elliott, Bruce Guthrie, Louisa Jorm, Alastair H. Leyland

**Affiliations:** 1 Health Economics Research Unit, University of Aberdeen, Foresterhill, Aberdeen, AB25 2ZD UK; 2 University of Lausanne, Lausanne, Switzerland; 3 University of Dundee, Dundee, UK; 4 UNSW Australia, Sydney, Australia; 5 University of Glasgow, Glasgow, UK

**Keywords:** Emergency admissions, Primary care, Ambulatory care sensitive conditions

## Abstract

**Background:**

Hospital admissions for Ambulatory Care Sensitive Conditions (ACSC) are those that could potentially be prevented by timely and effective disease management within primary care. ACSC admissions are increasingly used as performance indicators. However, key questions remain about the validity of these measures. The evidence to date has been inconclusive and limited to specific conditions. The aim of this study was to test the robustness of ACSC admissions as indicators of the quality of primary care. It is the first study to examine a wide range of ACSCs using longitudinal data which enables us to control for unmeasured characteristics which differ by practice but which are constant over time.

**Methods:**

Using longitudinal data at the practice level, from 907 Scottish practices for the time period 1/4/2005 to 31/32012, we explored the relationships between the quality of primary care, and hospital admissions for multiple ACSCs controlling for a wide range of covariates including characteristics of GP practices, characteristics of the practice population, hospital effects and year effects. We examined the impact of two dimensions of quality of care: clinical quality of and access to daytime general practice. Generalised Estimating Equations taking the form of Negative Binomial regression models with the practice population included as the exposure term were estimated.

**Results:**

We found that higher achievement on some clinical quality measures of primary care was associated with reduced ACSC emergency admissions. We also show that access to primary care was associated with ACSC emergency admissions. However, the effects were small and inconsistent and ACSC emergency admissions were associated with several confounding factors such as deprivation, rurality and distance to the hospital.

**Conclusions:**

The results suggest caution in the use of crude ACSC admission rates as a performance indicator of quality of primary care.

**Electronic supplementary material:**

The online version of this article (10.1186/s12875-019-0921-z) contains supplementary material, which is available to authorized users.

## Background

The steady increase in the rate of emergency admissions to hospitals is a major policy concern. It has been argued that a significant proportion of these admissions can be prevented by effective management and treatment in primary care. Conditions for which effective primary care management and treatment are expected to prevent hospital admissions are referred to as Ambulatory Care Sensitive Conditions (ACSC), which account for around 17% of all emergency admissions [1]. ACSC admissions are used as performance measures in the UK and internationally. They are for example used within the National Health Service (NHS) Outcome Framework Indicators in England and within the Health Improvement, Efficiency, Access to Services and Treatment (HEAT) targets in Scotland. ACSC admissions are particularly attractive as performance indicators as they can be straightforwardly generated from routine hospital data. However, key questions remain about the validity of these measures. For these indicators to be robust and useful performance measures it is crucial that they are actually attributable and sensitive to changes in quality of primary care.

Examining the relationship between quality of primary care and ACSC admissions is challenging. In most countries there are limited data on quality of primary care. The Quality and Outcomes Framework (QOF) introduced throughout the UK in 2004 provides measures of the clinical quality of primary care and thus provides a unique opportunity to investigate the relationship between primary care quality and ACSCs. The QOF was the world’s largest healthcare pay-for-performance programme, with > 10% of practice income dependent on performance measured by a range of indicators, primarily measures of chronic disease quality of care. Practice-level performance data is collected from electronic health records nationwide and publicly reported, providing a unique opportunity to investigate the relationship between primary care quality and ACSCs across an entire healthcare system. Furthermore, the introduction of patient experience surveys from 2008 onwards provides measures of access to daytime primary care which is another important quality dimension.

Quality measures are integral to the QOF and this has generated annual practice level data on a wide range of quality indicators. Previous studies have examined the relationship between practices’ performance on QOF indicators and ACSC admissions but have produced mixed results [2]. The majority of these studies used cross sectional analysis which may be confounded by unobserved factors that affect both admissions and quality of primary care and in several studies the sample size was small. Only four studies in England have used longitudinal data [3–6]. The key advantage of longitudinal analysis is that it can control for factors which have not been included in the analysis, remain unobserved (either because no measures of them exist or because the available measures are not judged sufficiently reliable) and which it is judged differ between practices but are likely to be constant over the time-frame of the analysis. ACSC admissions will be caused by a wide range of characteristics, some of which are not observed. Examples are characteristics of patients populations (for example, former mining villages have higher rates of admissions with respiratory problems because of dust diseases and smoking) and practice characteristics such as culture and these may be correlated with both ACSC admissions and quality of care. Longitudinal analysis can control for these unobserved characteristics by conditioning on baseline (pre-intervention) ACSC admissions conditioning on within practice correlation in unobserved factors associated with the outcome of interest that persists over time, or by using only within practice variation in outcomes and explanatory variables (fixed effects).

Previous longitudinal studies found that performance on QOF indicators was linked to ACSC admissions. However they focused on specific conditions and did not therefore examine the range of ACSC indicators. Among these studies Dusheiko et al. [3] found that higher achievement on QOF indicators linked to diabetes management were associated with reductions in emergency admissions for diabetes. Soljak et al. [4] also found better reported HbA1c control in the QOF to be associated with lower emergency and elective admissions for acute as well as long-term diabetes complications. Improvement in QOF achievement for dementia review was associated with reduced dementia and all ACSC emergency admissions, particularly in more deprived practices in England [5]. However Gutacker et al. [6] found that higher achievement on QOF indicators associated with mental health was associated with increases rather than decreases in psychiatric admissions. This difference in the direction of the effect across the different studies demonstrates the need to examine a range of ACSCs to understand whether there are any consistent and generalizable results in terms of the impact of achievement on QOF indicators on ACSC admissions. We identified no previously published studies that have combined longitudinal analysis with analysis of a range of conditions. Closest to our study is a recent paper in this journal by Busby et al. [7] which examined a range of factors associated with admissions for 28 ACSCs. However, their study was cross sectional and a general QOF measure rather than ACSC specific QOF measures were used to measure quality.

Access to primary care is another important dimension of quality of primary care. Several previous studies have found that patient reported measures, such as being able to make an appointment within 48 h, are associated with reduction in emergency admissions [8]. Cowling et al. [9] found more timely access to General Practice (GP) practices reduced self-referred emergency department visits in England. Again, the majority of these studies used cross sectional analysis and in several studies the sample size was small.

The proposition that ACSC admissions provide a measure of the quality of primary care therefore has to be explored over a wide range of ACSCs using longitudinal data. Only if there is a consistent relationship across a wide range of conditions can ACSC admissions be used with confidence to indicate the quality of primary care. This paper examines the relationship between the quality of primary care and hospital emergency admissions in Scotland for a wide range of chronic ACSCs (asthma; chronic obstructive pulmonary disease (COPD); diabetes complications; hypertension; angina; convulsions and epilepsy and stroke) using longitudinal analysis.

## Methods

The overall study design was a population-based retrospective analysis of routine data using multiple regression modelling. Annual ACSC emergency admissions at practice level were modelled as a function of clinical quality of care (QOF indicators) and access to care (patient experience measures). We used longitudinal data controlling for a wide range of covariates which may be correlated with admissions, quality indicators and access to primary care including composition of the practice population, hospital effects and baseline admission rates which is important if ACSC admissions are to be used as performance measures. The longitudinal data also allow us to control for unobserved characteristics which differ across practices but which are constant over time.

### Sample

Over the period 1/4/2005 to 31/3/2012 there were 1106 general practices in Scotland. The NHS provides universal coverage to all UK residents, with a requirement for registration with a single GP practice to access services. The analysis was restricted to practices with a list size of at least 1000. Smaller practices serve atypical populations, most commonly being either extremely remote or serving special populations such as homeless people. There are around 60 smaller practices. The number of practices included in the estimation ranged from 888 to 907 per year.

### The outcome variable: hospital admissions

The outcome variable was the annual number of ACSC emergency admissions at practice level. Hospital admissions data came from the Scottish Morbidity Records (SMR-01), and was collated by Information Services Division (ISD) of National Health Services Scotland (www.isdscotland.org). Those ACSCs which had an associated set of QOF incentivised indicators and were included in the NHS Scotland Potentially Preventable Admissions Indicator were initially selected for analysis. These were: asthma; chronic obstructive pulmonary disease (COPD); diabetes complications; hypertension; angina; and convulsions and epilepsy. Stroke was also included as this is incentivised within the QOF and identified as ACSC in the literature [6]. Admissions were identified by their International Statistical Classification of Diseases and Related Health Problems 10th Revision (ICD-10) codes as shown in Additional file 1. Patient admissions to hospital were aggregated to practice level in each financial year.

### Indicators of the clinical quality of disease management within primary care

Quality of disease management was measured using data reporting the practice’s achievement on QOF clinical indicators. These data were obtained from ISD. For the QOF indicators, “population achievement” rates on each QOF indicator were measured as the number of patients for whom an indicator is achieved divided by disease prevalence in the practice area. Only those indicators that were consistently defined over the time period and which might plausibly be related to ACSC admissions were selected for inclusion [10] and listed in Additional file 2. For example, the QOF indicator for asthma review was selected. These reviews are hypothesised to optimise asthma treatment, they are intended to improve asthma control and thus reduce ACSC asthma admissions. An example for angina is the percentage of patients with Coronary Heart Disease (CHD) whose last measured total cholesterol (measured in the last 15 months) is 5 mmol/l or less. Higher quality primary care should result in better controlled cholesterol in CHD patients and as a consequence reduce emergency admissions with angina.

Practices were able to exclude patients from the QOF indicators in a number of defined circumstances such as patient being deemed unsuitable for the treatment or being newly registered with the practice. For the purposes of this analysis, practice performance ignoring exception reporting was used as the measure of primary care quality. However, exception reporting rates were additionally included in the model to control for otherwise unmeasured differences in patient characteristics such as frailty and differences in reporting by practices.

### Quality of primary care measured in terms of access and continuity of care

Access to daytime primary care was measured using national patient experience surveys (http://www.gov.scot/Topics/Statistics/Browse/Health/GPPatientExperienceSurvey). These data are available from 2008 onwards. The patient experience surveys were sent to a random sample of patients registered with their GP in October of the relevant year. The total sample size across the 4 surveys is 629,495 and the average response rate was 33.5%. Survey weights were applied to make the estimates more representative of the practice population. Three patient experience measures that were hypothesised to be associated with rate of emergency admissions were included. Access to care was measured using: percentage of respondents who could see or speak to a doctor or nurse within 2 working days; and percentage of respondents able to book an appointment in advance. Continuity of care was measured using percentage of respondents who can usually see the doctor they prefer. Drive time to the nearest GP available was also included to explore whether ‘physical’ access also has an impact.

### Covariates

A number of covariates were included in the regression models to adjust for other practice factors which may be correlated with all of ACSC emergency admissions, quality indicators and access to primary care [5]. Some of these covariates may be confounders in that they affect both quality and ACSC emergency admissions independently. However, some of the covariates may be mediators in that they influence the quality of care which then affects ACSC emergency admissions. The latter is more likely with practice characteristics. The practice characteristics were: average age of GPs, percentage of female GPs, percentage of principal GPs, dispensing practice, type of contract the practice holds, size of the practice (number of patients), number of patients per GP, and prevalence rates for the conditions examined. These data were obtained from the Information Services Division (ISD). We also obtained data from ISD Scotland on the number of patients (by age group and gender registered with each practice in each datazone). Datazones are small areas which have between 500 and 1000 household residents (800 on average). We used these data to adjust for the age and gender mix of the patients and for a range of area based characteristics including deprivation measured by the 2009 Scottish Index of Multiple Deprivation (SIMD) [11] and remoteness and rurality measured by the Scottish government urban rural classification [12] using data obtained from the Scottish Neighbourhood Statistics website (http://statistics.gov.scot/).

Hospitals may differ in admission policies and in other characteristics which influence their propensity to admit. We adjusted for these hospital effects using the proportion of each practice’s emergency admissions at each hospital. This variable is analogous to including fixed hospital effects in the model to remove variation between hospitals in their ACSCs admission rates, independent of primary care quality, access, as well as patient morbidity and other risk factors. This adjusts for differential admission thresholds or other hospital dimensions such as capacity or quality (readmission rates) across hospitals. We adjusted for the accessibility of each hospital by taking the average distance from the patients’ datazone centroid to the nearest 5 acute hospitals [3].

Finally, dummy variables for the Community Health Partnership (CHP, which is an NHS Scotland locality organisation) that the practice belongs to were included to allow for geographic differences as well as variations in out of hours care provision, specialist disease services, etc.

A number of relevant policy developments took place over the time period including the establishment of the national Long Term Conditions Collaborative improvement programme and the introduction of a rapid ambulance protocol for acute stroke. We included a dummy variable for each financial year to control for these and other time dependent effects. Baseline rates (average admission rates over 2000/1 to 2003/4) were included to remove the effect of unobserved confounding practice factors that do not vary over time.

### Estimation method

Separate models were estimated for each ACSC. For each ACSC the corresponding disease specific QOF indicators were included. The dependent variable, number of annual admissions at practice level, is a count variable. Generalised Estimating Equations (GEE) taking the form of Negative Binomial regression models with the practice population included as the exposure term were estimated. GEE estimators account for the correlation structure of repeated observations from the same practices [13].

The annual number of ACSC emergency admissions at practice level was regressed on quality of disease management (QOF) indicators, access measures and the control covariates. It was assessed whether coefficients were statistically significant at a 5% level. Statistical significance at the 5% level implies that estimates would be either significantly above or below zero (the two-sided null hypothesis being not significantly different from zero) 95% of the time if we drew repeated samples from the population of GP practices and re-estimated the model.

### Sensitivity analysis

To explore sensitivity of results we also estimated practice fixed effects and practice random effects linear regression with practice ACSC rate as the dependent variable.

## Results

Population achievement on the QOF indicators was generally high but ranged from 54.8 for the percentage of patients with CHD who are treated with a beta blocker therapy to 96.3 for the percentage of patients with CHD whose notes have a record of blood pressure in the previous 15 months.

### Admission rates by practice

Table 1 shows the average hospital admission rates by primary care practice by ACSC. Rates were highest for COPD and they increased over the time period for diabetes complications and COPD but fell for angina and epilepsy. The descriptive statistics of the QOF indicators and covariates are reported in Additional files 3 and 4.

**Table 1:** Average Hospital Admission Rates by Practice (per 10,000 practice population)

	Asthma	COPD	Diabetes complications	Hyper-tension	Angina	Convulsions and Epilepsy	Stroke
2005/06	10.8	26.3	8.8	1.2	13.9	15.9	15.2
2006/07	13.1	28.9	9.1	1.1	12.7	16.0	15.0
2007/08	11.2	29.1	9.2	0.9	15.5	16.1	14.4
2008/09	12.3	32.0	9.1	0.9	13.9	16.7	15.0
2009/10	10.9	29.1	9.1	1.0	11.2	15.4	15.3
2010/11	10.7	31.0	9.6	1.0	10.5	14.1	15.1
2011/12	9.9	32.2	10.3	1.0	8.4	14.4	14.8

### Emergency admissions

**Fig. 1:**
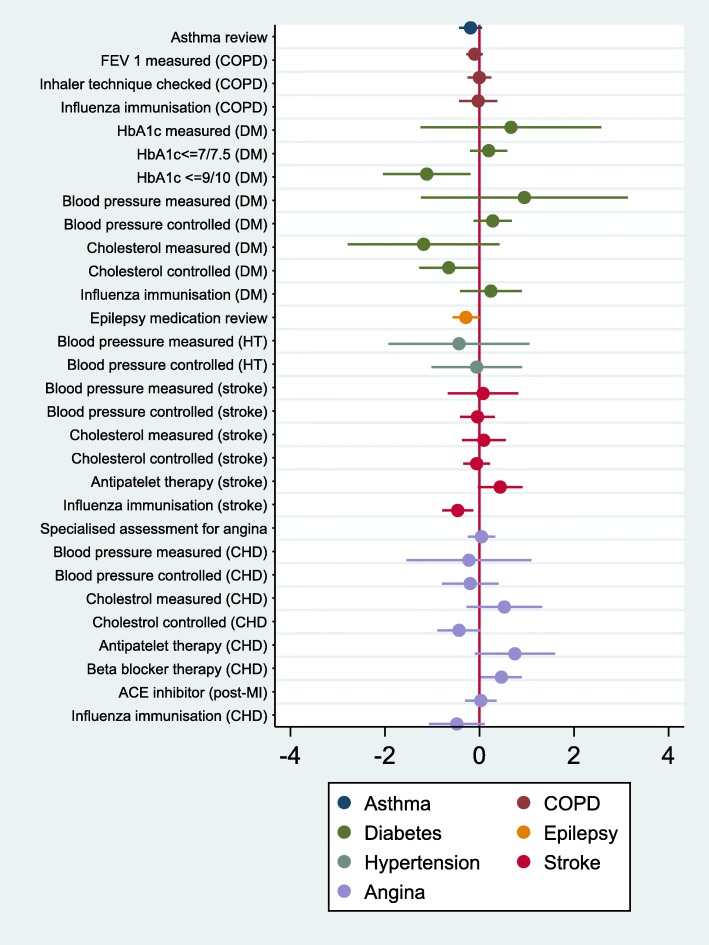
Relationship between clinical quality of primary care and ACSC emergency admissions. Coefficients and confidence intervals from GEE negative binomial regression models. The coefficients on the quality indicators show the change in % admissions associated with a 1% increase in population achievement. All models also include all covariates including Community Health Partnership dummies and proportions of practice admissions at each hospital

Figure 1 reports the results of the multivariate analysis for the QOF indicators. The full regression results are reported in Addition file 5. The results show that there is a statistically significant association (at a 5% level) between ACSCs and five QOF indicators of quality: total cholesterol controlled and HbA1c < =9/10 (diabetes complications), medication review (epilepsy), influenza immunisation (stroke) and betablocker therapy (angina). It should be noted that we are performing multiple significance tests and it is therefore possible that some of these significant effects are in fact not true effects.

Higher population achievement on the QOF indicators was associated with lower ACSCs on four out of the five indicators that were statistically significant at a 5% level. We also found the reverse on one of the significant indicators, higher population achievement on one QOF indicator associated with higher ACSCs, in the case of angina.

The magnitude of the relationship between quality improvement and emergency admissions were moderate. For example, a 10% increase in influenza immunisation for patients with stroke was associated with a 4.6% decrease in ACSC emergency admissions for stroke. On average, 78% of patients with TIA or stroke received influenza immunisation between September 2010 and March 2011. Achieving a 10% increase to 88% will result in an annual reduction of around 370 emergency stroke admissions in total across Scotland when evaluated at the mean of the sample.

For the majority of the QOF indicators of quality there is no statistically significant association with ACSC admissions. Moreover, none of the QOF indicators are associated with ACSC admissions for asthma, COPD and hypertension. It is also interesting to compare indicators that were similar across several conditions such as blood pressure (measured or controlled), cholesterol (measured or controlled) and influenza vaccination. There was no significant association between quality indicators for blood pressure (measured or controlled) and ACSC admissions for diabetes complications, hypertension or angina. A significant association was found for stroke. There was no significant association between quality indicators for cholesterol (measured or controlled) and ACSC admissions for stroke and angina. A significant association was found for diabetes complications. Influenza immunisation was not associated with COPD, diabetes complications. A significant association was found for stroke. Similar indicators therefore do not seem to have a consistent effect across all ACSC admissions.

**Fig. 2:**
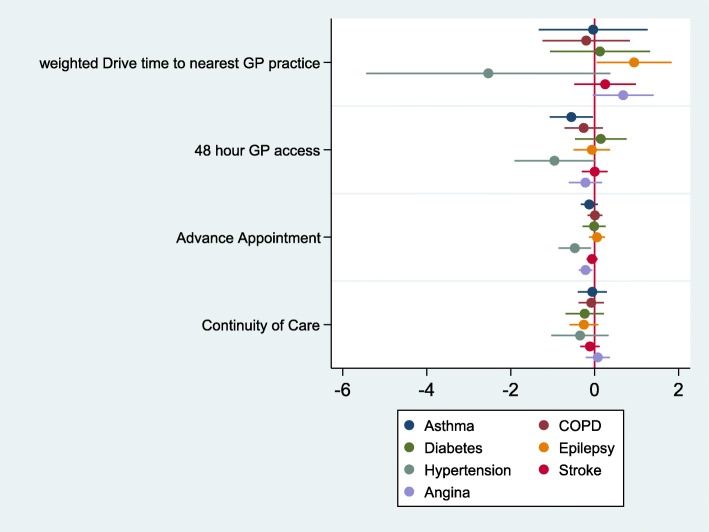
Relationship between access and ACSC emergency admissions. Coefficients and confidence intervals from GEE negative binomial regression models. The coefficients on the access measures show the change in % admissions associated with a 1% increase in population achievement. All models also include all covariates including Community Health Partnership dummies and proportions of practice admissions at each hospital

Figure 2 reports the results of the multivariate analysis for the access measures. One or more of the access measures from the patient experience surveys were significantly associated (at a 5% level) with admissions for three conditions: asthma, hypertension and angina. Longer drive times to nearest GP practice was associated with higher admissions for convulsions and epilepsy. Effect sizes for 48 h access were more important for asthma and hypertension with 10% improvements in access associated with 5.6 and 9.6% reductions on admissions, but effects for advanced booking were smaller. For example, a 10% increase in being able to book an advanced appointment was associated with a 2.2% decrease in angina admissions. Note that the average percentage of patients being able to book in advance was already relatively high, namely 84.8%.

The full model results are available in Additional file 5. Prevalence rates and baseline admission rates were significantly associated (at a 5% level) with increases in ACSC emergency admissions. Area level education deprivation was significantly associated (at a 5% level) with increases in admissions for 4 out of 7 conditions. Having a higher proportion of the practice population living in remote and rural areas was significantly associated (at a 5% level) with decreases in admissions for 3 out of 7 conditions. Further distance to hospital was associated with a decrease in admissions for 3 out of 7 conditions. A higher proportion of female GPs was significantly associated (at a 5% level) with decreases in admissions for diabetes complications. Practices with larger populations had fewer ACSC emergency admissions for 2 out of 7 conditions. GMS practices had lower admissions for angina. The admission rate was 6.8% lower for GMS practices. Asthma and COPD admissions indicated significant upward trends in later years, while hypertension and stroke showed significant downward trends with the other conditions also showing reductions on emergency admissions in later years.

### Sensitivity analyses

The consistency of the results of the emergency admissions models across the different regression techniques was examined. The sign of the coefficients was consistent across all estimation methods for those coefficients that were statistically significant at a 5% level in the base case model. The significance level varied for some of the indicators (Additional file 6).

## Discussion

### Main findings

This study examined the relationship between quality and accessibility of primary care and admissions across a range of ACSCs in Scotland. The results showed that only a small number of measures of the clinical quality of primary care is associated with reduced ACSC emergency admissions. The majority of the quality measures were not statistically significant and for three ACSC (asthma, COPD, and hypertension) none of the quality measures were significant. There are two possible explanations, which are not mutually exclusive. First, that ACSC admissions are not actually markers of ambulatory care quality. Second, that the quality indicators underpinning the QOF pay-for-performance programme are not actually good measures of primary care quality.

We also found associations in the opposite direction, with higher achievement on clinical quality associated with increased ACSC emergency admissions, for some of the measures. There are a number of possible explanations for the positive association: it may reflect confounding by indication (more intensive management of more severely ill patients); medication and blood pressure measurement may increase patients’ awareness of their ill-health leading to higher number of emergency admissions.

In terms of access of care, we found that patient experience measures were associated with reduction in ACSC emergency admissions for three conditions. Higher percentage of practice patients being able to see or speak to a doctor or nurse within 2 working dates or able to book an appointment in advance the lower the ACSC admissions. There was no association between patient experience of continuity of care and ACSC admission rates. Drive times to nearest GP practice was significant for one ACSC only showing that longer drive times were associated with higher admissions.

### Strength and limitations of the study

The main strength of this study is that it examined several chronic ACSCs over a relatively long time period using robust analysis. This allowed comparisons to be made across different conditions. It utilised the relatively rich data available on quality of primary care in the UK. Moreover, admissions data from Scotland are recognised to be of consistently higher quality than most other countries. However, whilst the QOF provides data on many quality indicators it is also recognised that practices were generally performing at a high level. There was relatively little variation between practices in these measures and it could also be argued that they indicate more the ability to meet targets than to provide high quality care. Whilst data were available on a wide range of potential factors associated with ACSCs, there are likely to remain several unobserved factors given the real world complexity around healthcare, including factors like patient health literacy, patient choice, other social determinants of patients and supply side factors [14]. The analysis used seeks to control for factors like these which are time-invariant over the timescale of this analysis, but like all observational studies it is not possible to account for all possible confounders.

### Comparison with other studies

Achievements on QOF indicators were not significantly associated with emergency admissions in the majority of previous studies [2]. However, most previous studies used cross-sectional data. Using longitudinal data and similar methods of analysis, Dusheiko et al. [3], Soljak et al. [4], Kasteridis et al. [5] and Guttacker et al. [6] and also found that performance on QOF indicators is associated with ACSC emergency admissions in England. This suggests that longitudinal data are better able to identify the effect of clinical quality as these data can control for unobserved factors that may be associated with ACSC admissions and that persist over time. Our results in terms of access of care are in line with the previous studies which also found that that patient reported measures, such as being able to make an appointment within 48 h, are associated with reduction in emergency admissions [8].

The results of this study also showed that deprivation, rurality, distance to hospital, size of practice population were associated with ACSC emergency admissions. In a systematic review, Purdy and Huntly [8] show that age and deprivation are the strongest risk factors for ACSC admissions. They also show that ethnicity, deprivation, distance to hospital, rurality, lifestyle, access, size of practice population and meteorological factors are all associated with ACSC admissions. We were unable to control for lifestyle, ethnicity and meteorological factors but found that the results of our study in terms of deprivation, rurality, distance to hospital and size of practice population were in line with the literature. Having a higher proportion of the practice population living in remote and rural areas, in areas of low education deprivation or further away from a hospital was associated with lower ACSC admissions. Practices with larger populations had fewer ACSC admissions.

These studies and our own establish that ACSCs are to some extent sensitive to the quality and accessibility of primary care. This would support their use as a quality indicator or performance measure across health care providers or healthcare administrative areas as they are used in the United States for Accountable Care Organisations (ACOs), in NHS England for Clinical Commissioning Groups (CCGs) as well as in other countries such as Australia and Canada. However, there may be less validity in the comparison of absolute differences in crudely standardised rates, given the significance of economic, social circumstances of patients as well as the proximity and influence of specialist hospital providers. The rare nature of many of the indicated ACSCs and sensitivity to random or unavoidable variations due to changes in the environment (e.g. flu epidemics, climatic variability etc.) could lead to significant differences in observed ACSC performance. Hence their use as an indicator requires measurement and comparison over a number of years, with adjustment for baseline differences and a rich set of confounding factors that vary over time. This need for adjustment has been previously recognised but is not often performed which may in part be due to a lack of robust data and methodology [15]. Some attempts have been made to partially account for some of the factors. In Australia for example rates are presented by rurality and socioeconomic status [16]. Our study and other studies suggest that further efforts should be made to adjust the indicators for a wider range of factors.

### Meaning of the study for clinicians and policy makers

That performance on some of the QOF indicators was associated with ACSC emergency admissions suggests that ACSC admission rates do, to at least some degree, reflect primary care quality. However, the effects were small and inconsistent. ACSC emergency admissions were more consistently associated with other factors outside the control of primary care. There is evidence that disease management programmes can reduce emergency hospital admissions but linking crude ACSC admission rates to performance should be treated with considerable caution when used as a measure of primary care quality. Adjusting ACSC admission rates for confounding factors such as age, deprivation, rurality, as well as proximity to specific hospitals of the practice population would improve its utility as a performance measure of quality of primary care.

### Unanswered questions and future research

Purdy and Huntley [8] and more recent evidence from Australia (Falster et al. [17]) suggest that individual characteristics, in particular individual’s lifestyles, explained a relatively large part of the variation in ACSC emergency admissions. Examining the role of lifestyle factors in ACSC emergency admissions in Scotland and the rest of the UK would be a fruitful line of future enquiry. The definition of ACSCs was based on the NHS Potentially Preventable Admissions Indicator. It would be interesting to explore how sensitive the results are to changing the definition of ACSCs.

## Conclusions

This research showed that higher achievement in some measures of the clinical quality of primary care and better access to care is associated with reduced ACSC admissions. However, the effects were small and inconsistent and ACSC emergency admissions were associated with several confounding factors such as deprivation, rurality and distance to the hospital. The results of this research suggest caution in the use of crude ACSC admission rates as an indicator of primary care performance.

## Additional files

Additional file 1: ICD-10 coding used to define ACSC emergency admissions. A list of the ICD-10 coding used to define the ACSC emergency admissions. (DOCX 14 kb)

Additional file 2: QOF indicators. Description of the QOF indicators included in the analysis. (DOCX 16 kb)

Additional file 3: Descriptive statistics covariates. Descriptive statistics of the covariates included in the analysis. (DOCX 16 kb)

Additional file 4: Quality of disease management indicators. Descriptive statistics of the QOF indicators. (DOCX 19 kb)

Additional file 5: Full regression results. Full regression results including the covariates. (DOCX 34 kb)

Additional file 6: Robustness checks - sign and significance level of coefficients. Robustness checks of the results. (DOCX 27 kb)

## Abbreviations

ACO: Accountable Care Organisations
ACSC: Ambulatory Care Sensitive Conditions
CHD: Coronary Heart Disease
CHP: Community Health Partnership
COPD: Chronic Obstructive Pulmonary Disease
GCG: Clinical Commissioning Groups
GEE: Generalised Estimating Eqs.
GP: General Practice
HEAT: Health Improvement, Efficiency, Access to Services and Treatment
ICD-10: International Statistical Classification of Diseases and Related Health Problems 10th Revision
ISD: Information Services Division
NHS: National Health Service
QOF: Quality and Outcomes Framework
SIMD: Scottish Index of Multiple Deprivation
SMR-01: Scottish Morbidity Records
