# Supplementary material for: The impact of quality and accessibility of primary care on emergency admissions for a range of chronic ambulatory care sensitive conditions (ACSCs) in Scotland: longitudinal analysis
Source: BMC Fam Pract. 2019 Feb 22;20:32. doi: 10.1186/s12875-019-0921-z (PMC6385424; doi:10.1186/s12875-019-0921-z)
Supplement: Supplementary file 1 — ICD-10 coding used to define ACSC emergency admissions. A list of the ICD-10 coding used to define the ACSC emergency admissions. (DOCX 14 kb) [file 12875_2019_921_MOESM1_ESM.docx]

**Additional file 1. ICD-10 coding used to define ACSC emergency admissions**

| **Asthma** | | |
| --- | --- | --- |
| J45 | Asthma | Principal diagnosis |
| J46 | Status asthmaticus | Principal diagnosis |
| **COPD** | | |
| J20 | Acute bronchitis | Principal diagnosis. Exclude episodes with following main ops K01 - K50, K56, K60 - K61 |
| J41 | Simple and mucopurulent chronic bronchitis | Principal diagnosis. Exclude episodes with following main ops K01 - K50, K56, K60 - K61 |
| J42 | Unspecified chronic bronchitis | Principal diagnosis. Exclude episodes with following main ops K01 - K50, K56, K60 - K61 |
| J43 | Emphysema | Principal diagnosis. Exclude episodes with following main ops K01 - K50, K56, K60 - K61 |
| J44 | Other chronic obstructive pulmonary disease | Principal diagnosis. Exclude episodes with following main ops K01 - K50, K56, K60 - K61 |
| J47 | Bronchiectasis | Principal diagnosis. Exclude episodes with following main ops K01 - K50, K56, K60 - K61 |
| **Diabetes complications** | | |
| E10; .0 - .8 | Type 1 diabetes mellitus | Any diagnosis |
| E11; .0 - .8 | Type 2 diabetes mellitus | Any diagnosis |
| E12; .0 - .8 | Malnutrition-related diabetes mellitus | Any diagnosis |
| E13; .0 - .8 | Other specified diabetes mellitus | Any diagnosis |
| E14; .0 - .8 | Unspecified diabetes mellitus | Any diagnosis |
| **Convulsions and epilepsy^1^** | | |
| G40 | Epilepsy | Principal diagnosis |
| G41 | Status epilepticus | Principal diagnosis |
| R56 | Convulsions, not elsewhere specified | Principal diagnosis |
| **Hypertension** | | |
| I10 | Essential (primary) hypertension | Principal diagnosis. Exclude episodes with following main ops K01 - K50, K56, K60 - K61 |
| I11.9 | Hypertensive heart disease without (congestive) heart failure | Principal diagnosis. Exclude episodes with following main ops K01 - K50, K56, K60 - K61 |
| **Stroke** | | |
| I60 | Subarachnoid hemorrhage | Principal diagnosis |
| I61 | Intracerebral hemorrhage | Principal diagnosis |
| I63 | Cerebral infarction | Principal diagnosis |
| I64 | Stroke, not specified as hemorrhage or infarction | Principal diagnosis |
| I66 | Occlusion and stenosis of cerebral arteries, not resulting in cerebral infarction | Principal diagnosis |
| I67.2 | Other cerebrovascular diseases. Cerebral atherosclerosis | Principal diagnosis |
| I69.8 | Sequelae of other and unspecified cerebrovascular diseases | Principal diagnosis |
| R47.0 | Speech disturbances, not elsewhere classified Dysphasia and aphasia | Principal diagnosis |
| **Angina** | | |
| I20 | Angina pectoris | Principal diagnosis. Exclude episodes with main ops K40 K45 K49 K60 K65 K66 |

^1^ eclampsia (O15) was excluded as this is a pregnancy related condition and not incentivised within the QOF
