# Supplementary material for: The impact of quality and accessibility of primary care on emergency admissions for a range of chronic ambulatory care sensitive conditions (ACSCs) in Scotland: longitudinal analysis
Source: BMC Fam Pract. 2019 Feb 22;20:32. doi: 10.1186/s12875-019-0921-z (PMC6385424; doi:10.1186/s12875-019-0921-z)
Supplement: Supplementary file 2 — QOF indicators. Description of the QOF indicators included in the analysis. (DOCX 16 kb) [file 12875_2019_921_MOESM2_ESM.docx]

**Additional file 2. QOF indicators**

|  | **Description** | **Changes** |
| --- | --- | --- |
| **Asthma** |  |  |
| Asthma review | The percentage of patients with asthma who have had an asthma review in the last 15 months |  |
| **Hypertension** |  |  |
| Blood pressure measured  (HT) | The percentage of patients with hypertension in which there is a record of the blood pressure in the past 9 months |  |
| Blood pressure controlled (HT) | The percentage of patients with hypertension in whom the last blood pressure (measured in last 9 months) is 150/90 or less |  |
| **Stroke** |  |  |
| Blood pressure measured (stroke) | The percentage of patients with TIA or stroke who have a blood pressure in the notes in the preceding 15 months | Deleted in 2010/11 |
| Blood pressure controlled (stroke) | The percentage of patients with a history of TIA or stroke in whom the last blood pressure reading (measured in the last 15 months) is 150/90 or less |  |
| Total cholesterol measured (stroke) | The percentage of patients with TIA or stroke who have a record of total cholesterol (measured in the last 15 months) in the last 15 months |  |
| Total cholesterol controlled stroke) | The percentage of patients with TIA or stroke whose last measured total cholesterol (measured in the last 15 months) is 5mmol/l or less |  |
| Antiplatelet therapy (stroke) | The percentage of patients with a stroke shown to be non-haemorrhagic, or a history of TIA, who have a record that aspirin, an alternative anti-platelet therapy, or an anti-coagulant is being taken (unless a contraindication or side effects are recorded) | In 2006/07: anti-platelet specified: aspirin, clopidogrel, dipyridamole or a combination |
| Influenza immunisation (stroke) | The percentage of patients with TIA or stroke who have had influenza immunisation in the preceding 1 September to 31 March |  |
| **COPD** |  |  |
| FEV1 measured (COPD) | The percentage of patients with COPD with a record of FeV1 in the previous 27 months | In 2006/07 changed to 15 months |
| Inhaler technique checked (COPD) | The percentage of patients with COPD receiving inhaled treatment in whom there is a record that inhaler technique has been checked in the preceding 2 years | In 2006/07 changes to previous 15 months  In 2009/10 changes to: the percentage of patients with COPD who have had a review , undertaken by a healthcare professional, including an assessment of breathlessness using the MRC dyspnoea score in the preceding 15 months |
| Influenza immunisation (COPD) | The percentage of patients with COPD who have had influenza immunisation in the preceding 1 September to 31 March |  |
| **Epilepsy** |  |  |
| Epilepsy medication review | The percentage of patients age 16 and over on drug treatment for epilepsy who have a record of medication review in the previous 15 months | In 2006/07 changed to age 18 and specified that medication review involves patient and/or carer |
| **Angina** |  |  |
| Specialist assessment for newly diagnosed angina | The percentage of patients with newly diagnosed angina (diagnosed after 01/04/03) who are referred for exercise testing and/or specialist assessment | In 2011/12 date changed to after 01/04/11 and referred for specialist assessment in 2011 |
| Blood pressure measured (CHD) | The percentage of patients with Coronary Heart Disease (CHD) whose notes have a record of blood pressure in the previous 15 months | Deleted in 2011/12 |
| Blood pressure controlled (CHD) | percentage of patients with CHD, in whom the last blood pressure reading (measured in the last 15 months) is 150/90 or less |  |
| Cholesterol measured (CHD) | The percentage of patients with CHD whose notes have a record of total cholesterol in the previous 15 months | Deleted in 2011/12 |
| Cholesterol controlled (CHD) | The percentage of patients with CHD whose last measured total cholesterol (measured in the last 15 months) is 5mmol/l or less |  |
| Antiplatelet therapy (CHD) | The percentage of patients with CHD with a record in the last 15 months that aspirin, an alternative anti-platelet therapy, or an anti-coagulant is being taken (unless a contraindication or side effects are recorded) |  |
| Beta blocker therapy (CHD) | The percentage of patients with CHD who are currently treated with a beta blocker (unless a contraindication or side effects are recorded |  |
| ACE inhibitor therapy (post-MI) | The percentage of patients with a history of myocardial infarction (diagnosed after 1April 2003) who are currently treated with an ACE inhibitor | In 2011/12 date changed to after 1April 2011 and currently treated with an ACE inhibitor (or ARB if ACE intolerant), aspirin or an alternative anti-platelet therapy, beta-blocker and statin (unless a contraindication or side effects are recorded) |
| Influenza immunisation CHD | The percentage of patients with CHD who have had influenza immunisation in the preceding 1 September to 31 March |  |
| **Diabetes** |  |  |
| HbA1c measured (DM) | The percentage of diabetic patients who have a record of HbA1c or equivalent in the previous 15 months | Deleted in 2011/12 |
| HbA1c ≤ 7/7.5 (DM) | The percentage of patients with diabetes in whom the last HbA1c is 7.4 or less (or equivalent test/ reference range depending on local laboratory) in the last 15 months | In 2006/07 changed to 7.5 or less. In 2009/10 changed to 7 or less. In 2011/12 changed to the last IFCC- HbA1 is 59mmol/mol (equivalent to HbA1 of 7.5% in DCCT values) or less |
| HbA1c ≤ 9/10 (DM) | The percentage of patients with diabetes in whom the last HbA1c is 10 or less (or equivalent test/ reference range depending on local laboratory) in the last 15 months | In 2009/10 changes to 9 or less. In 2011/12 changed to the last IFCC- HbA1 is 75mmol/mol (equivalent to HbA1 of 9% in DCCT values) or less |
| Blood pressure measured (DM) | The percentage of patients with diabetes who have a record of the blood pressure in the previous 15 months | Deleted in 2011/12 |
| Blood pressure controlled (DM) | The percentage of patients with diabetes in whom the last blood pressure is 145/85 or less |  |
| Cholesterol measured (DM) | The percentage of patients with diabetes who have a record of total cholesterol in the previous 15 months | Deleted in 2011/12 |
| Cholesterol controlled (DM) | The percentage of patients with diabetes whose last measured total cholesterol within the previous 15 months is 5mmol/l or less |  |
| Influenza immunisation (DM) | The percentage of patients with diabetes who have had influenza immunisation in the preceding 1 September to 31 March |  |
