# Supplementary material for: The impact of quality and accessibility of primary care on emergency admissions for a range of chronic ambulatory care sensitive conditions (ACSCs) in Scotland: longitudinal analysis
Source: BMC Fam Pract. 2019 Feb 22;20:32. doi: 10.1186/s12875-019-0921-z (PMC6385424; doi:10.1186/s12875-019-0921-z)
Supplement: Supplementary file 3 — Descriptive statistics covariates. Descriptive statistics of the covariates included in the analysis. (DOCX 16 kb) [file 12875_2019_921_MOESM3_ESM.docx]

**Additional file 3. Descriptive statistics covariates**

| **Name** | **Description** | **Mean** | **SD** |
| --- | --- | --- | --- |
| 48 hour GP access | The percentage of patients able to book an appointment 48 hours in advance | 93.8 | 5.2 |
| Advance Appointment | The percentage of patients able to book an appointment 2+days in advance | 84.8 | 14.2 |
| Continuity of care | The percentage of patients being able to book appointment with preferred doctor | 87.2 | 9.9 |
| Out of hours care rating | The percentage of patients who rate out of hours care as good/excellent | 71.9 | 3.0 |
| Drive time to nearest GP practice | Driving time to the nearest GP practice for practice population | 3.50 | 2.9 |
| Practice population | List size | 5618 | 3250.8 |
| Practice population per GP | Practice population per GP | 1243 | 466.5 |
| GMS practice | Whether the practice is a GMS practice | 0.91 | 0.29 |
| Physician age | Average age of GPs at the practice | 45.25 | 5.8 |
| Female physicians | Proportion of female GPs at the practice | 0.468 | 0.25 |
| GP principals | Proportion of GP principals at the practice | 0.831 | 0.22 |
| Dispensing practice | Whether the practice is a dispensing practice | 0.088 | 0.28 |
| Male 0-4(%) | Proportion of male and female patients by age group | 0.026 | 0.01 |
| Male 5-14(%) | Proportion of male and female patients by age group | 0.055 | 0.01 |
| Male 15-24(%) | Proportion of male and female patients by age group | 0.064 | 0.02 |
| Male 25-44(%) | Proportion of male and female patients by age group | 0.148 | 0.04 |
| Male 45-64(%) | Proportion of male and female patients by age group | 0.135 | 0.02 |
| Male 65-74(%) | Proportion of male and female patients by age group | 0.041 | 0.01 |
| Male 75-84(%) | Proportion of male and female patients by age group | 0.022 | 0.01 |
| Male 85+(%) | Proportion of male and female patients by age group | 0.005 | 0.00 |
| Female 0-4(%) | Proportion of male and female patients by age group | 0.025 | 0.01 |
| Female 5-14(%) | Proportion of male and female patients by age group | 0.053 | 0.01 |
| Female 15-24(%) | Proportion of male and female patients by age group | 0.061 | 0.03 |
| Female 25-44(%) | Proportion of male and female patients by age group | 0.139 | 0.03 |
| Female 45-64(%) | Proportion of male and female patients by age group | 0.132 | 0.02 |
| Female 65-74(%) | Proportion of male and female patients by age group | 0.047 | 0.12 |
| Female 75-84(%) | Proportion of male and female patients by age group | 0.033 | 0.01 |
| Female 85+(%) | Proportion of male and female patients by age group | 0.013 | 0.01 |
| Income rate | Income domain 2009 rate for practice population | 16.1 | 7.3 |
| Education score | Education, Skills and Training domain 2009 score for practice population | 0.034 | 0.62 |
| Housing score | Mean Housing domain 2009 score for practice population | 21.1 | 11.6 |
| Crime rank | Crime domain 2009 rank weighted for practice population | 3255 | 1067.1 |
| Employment rate | Employment domain 2009 rate weighted for practice population | 12.3 | 5.3 |
| Large Urban Areas | Proportion of practice population living in Large Urban areas | 0.405 | 0.48 |
| Other Urban Areas | Proportion of practice population living in Other Urban Areas, Settlements of 10,000 to 125,000 people | 0.249 | 0.40 |
| Accessible Small Towns | Proportion of practice population living in Accessible Small Towns, Settlements of between 3,000 and 10,000 people and within 30 minutes drive of a settlement of 10,000 or more | 0.076 | 0.21 |
| Remote Small Towns | Proportion of practice population living in Remote Small Towns, Settlements of between 3,000 and 10,000 people and with a drive time of over 30 minutes to a settlement of 10,000 or more | 0.036 | 0.15 |
| Accessible Rural | Proportion of practice population living in Accessible Rural, Areas with a population of less than 3,000 people, and within a 30 minute drive time of a settlement of 10,000 or more | 0.126 | 0.23 |
| Remote Rural | Proportion of practice population living in Remote Rural**,** Areas with a population of less than 3,000 people, and with a drive time of over 30 minutes to a settlement of 10,000 or more | 0.108 | 0.28 |
| Distance to nearest 5 hospitals | Mean distance to nearest 5 hospitals for practice population | 17.9 | 0.05 |
