# Supplementary material for: The impact of quality and accessibility of primary care on emergency admissions for a range of chronic ambulatory care sensitive conditions (ACSCs) in Scotland: longitudinal analysis
Source: BMC Fam Pract. 2019 Feb 22;20:32. doi: 10.1186/s12875-019-0921-z (PMC6385424; doi:10.1186/s12875-019-0921-z)
Supplement: Supplementary file 4 — Quality of disease management indicators. Descriptive statistics of the QOF indicators. (DOCX 19 kb) [file 12875_2019_921_MOESM4_ESM.docx]

**Additional file 4. Quality of disease management indicators**

|  | **2005/06** | | **2006/07** | | **2007/08** | | **2008/09** | | **2009/10** | | **2010/11** | | **2011/12** | |
| --- | --- | --- | --- | --- | --- | --- | --- | --- | --- | --- | --- | --- | --- | --- |
|  | **Mean** | **SD** | **Mean** | **SD** | **Mean** | **SD** | **Mean** | **SD** | **Mean** | **SD** | **Mean** | **SD** | **Mean** | **SD** |
| **Asthma** | | | | | | | | | | | | | | |
| Asthma review | 74.9 | 11.8 | 74.5 | 10.6 | 75.0 | 10.4 | 74.6 | 10.3 | 73.9 | 10.3 | 72.9 | 10.3 | 71.8 | 10.6 |
| Prevalence | 5.4 | 1.2 | 5.5 | 1.2 | 5.5 | 1.2 | 5.7 | 1.2 | 5.9 | 1.3 | 6.0 | 1.3 | 6.1 | 1.3 |
| **Hypertension** | | | | | | | | | | | | | | |
| Blood pressure measured (HT) | 91.5 | 5.2 | 91.6 | 4.7 | 91.7 | 3.9 | 91.7 | 3.7 | 91.2 | 4.1 | 90.7 | 4.1 | 90.7 | 4.0 |
| Blood pressure controlled (HT) | 74.9 | 8.2 | 76.3 | 7.4 | 77.7 | 6.8 | 78.5 | 6.4 | 78.5 | 6.2 | 78.0 | 6.2 | 78.1 | 5.9 |
| Prevalence | 12.5 | 3.0 | 12.9 | 3.1 | 13.2 | 3.1 | 13.5 | 3.1 | 13.7 | 3.1 | 13.9 | 3.2 | 14.1 | 3.2 |
| **Angina** | | | | | | | | | | | | | | |
| Specialist assessment for newly diagnosed angina | 84.3 | 13.9 | 87.0 | 11.1 | 88.6 | 9.5 | 89.9 | 8.4 | 90.3 | 8.2 | 90.1 | 8.2 | 85.3 | 16.2 |
| Blood pressure measured (CHD) | 95.6 | 3.3 | 96.0 | 3.2 | 96.1 | 2.7 | 96.3 | 2.6 | 96.3 | 2.8 | 95.9 | 2.8 |  |  |
| Blood pressure controlled (CHD) | 85.6 | 6.2 | 87.0 | 5.7 | 87.5 | 5.2 | 88.3 | 4.9 | 88.5 | 5.1 | 88.0 | 5.1 | 87.5 | 5.0 |
| Cholesterol measured (CHD) | 90.5 | 5.7 | 90.9 | 5.3 | 91.5 | 4.4 | 91.8 | 4.2 | 91.9 | 4.4 | 91.4 | 4.4 |  |  |
| Cholesterol controlled (CHD) | 71.9 | 8.7 | 74.6 | 7.5 | 75.7 | 6.9 | 75.8 | 6.1 | 76.1 | 6.3 | 75.3 | 6.3 | 73.1 | 6.9 |
| Antiplatelet therapy (CHD) | 92.1 | 4.0 | 92.4 | 3.7 | 92.7 | 3.3 | 92.9 | 3.0 | 92.7 | 3.1 | 92.2 | 3.1 | 92.0 | 3.2 |
| Beta blocker therapy (CHD) | 54.8 | 7.0 | 55.2 | 7.1 | 56.2 | 6.9 | 57.3 | 6.7 | 58.3 | 6.4 | 59.2 | 6.4 | 60.5 | 6.3 |
| ACE inhibitor therapy (CHD) | 79.1 | 10.9 | 81.3 | 10.3 | 81.8 | 9.3 | 82.0 | 8.6 | 81.6 | 7.5 | 81.1 | 7.5 | 69.1 | 15.4 |
| Influenza immunisation (CHD) | 80.5 | 6.6 | 79.1 | 7.0 | 78.9 | 6.9 | 80.2 | 6.4 | 81.3 | 5.9 | 81.5 | 5.9 | 82.0 | 5.7 |
| Prevalence | 4.6 | 1.2 | 4.6 | 1.2 | 4.6 | 1.2 | 4.5 | 1.2 | 4.5 | 1.1 | 4.5 | 1.1 | 4.5 | 1.1 |
| **COPD** | | | | | | | | | | | | | | |
| FEV1 measured (COPD) | 76.9 | 16.8 | 74.7 | 14.8 | 76.6 | 13.0 | 77.5 | 11.5 | 77.9 | 12.4 | 76.5 | 12.4 | 75.4 | 12.1 |
| Inhaler technique checked (COPD) | 85.7 | 12.0 | 82.8 | 11.2 | 84.4 | 9.6 | 85.2 | 8.8 | 78.2 | 10.5 | 80.7 | 10.5 | 80.4 | 9.5 |
| Influenza immunisation (COPD) | 80.9 | 8.2 | 79.0 | 8.4 | 78.4 | 8.1 | 79.7 | 7.7 | 81.5 | 7.2 | 81.4 | 7.2 | 81.6 | 6.8 |
| Prevalence | 1.9 | 1.1 | 2.0 | 1.0 | 2.0 | 1.0 | 2.0 | 1.0 | 2.1 | 1.0 | 2.2 | 1.0 | 2.2 | 1.0 |
| **Diabetes complications** | | | | | | | | | | | | | | |
| HbA1c measured (DM) | 95.1 | 3.3 | 94.9 | 3.3 | 94.9 | 3.2 | 95.1 | 3.1 | 95.3 | 3.1 | 95.4 | 3.1 |  |  |
| HbA1c ≤ 7/7.5 (DM) | 51.9 | 10.3 | 56.9 | 8.3 | 57.7 | 8.0 | 57.7 | 7.7 | 45.8 | 8.5 | 46.7 | 8.5 | 59.3 | 8.2 |
| HbA1c ≤ 9/10 (DM) | 86.5 | 5.3 | 87.0 | 5.0 | 87.0 | 4.7 | 87.2 | 4.7 | 80.5 | 6.1 | 80.8 | 6.1 | 80.2 | 6.3 |
| Blood pressure measured (DM) | 96.9 | 2.4 | 97.1 | 2.1 | 96.8 | 2.3 | 96.8 | 2.3 | 96.6 | 2.6 | 96.4 | 2.6 |  |  |
| Blood pressure controlled (DM) | 73.4 | 9.8 | 75.6 | 8.8 | 76.3 | 8.6 | 77.1 | 7.9 | 77.6 | 8.1 | 77.3 | 8.1 |  |  |
| Cholesterol measured (DM) | 94.1 | 3.7 | 94.1 | 3.7 | 94.1 | 3.4 | 94.2 | 3.4 | 94.0 | 3.7 | 93.7 | 3.7 |  |  |
| Cholesterol controlled (DM) | 73.3 | 8.0 | 76.0 | 6.6 | 76.4 | 6.2 | 76.2 | 6.1 | 76.1 | 5.8 | 75.4 | 5.8 | 74.2 | 5.9 |
| Influenza immunisation (DM) | 77.6 | 7.3 | 75.1 | 7.6 | 75.1 | 7.6 | 75.9 | 6.9 | 78.6 | 6.5 | 77.9 | 6.5 | 78.4 | 6.3 |
| Prevalence | 3.5 | 0.8 | 3.6 | 0.8 | 3.8 | 0.8 | 4.0 | 0.9 | 4.2 | 0.9 | 4.4 | 0.9 | 4.5 | 1.0 |
| **Convulsions and epilepsy** | | | | | | | | | | | | | | |
| Epilepsy medication review | 90.5 | 9.5 | 90.2 | 8.6 | 89.6 | 9.3 | 89.9 | 9.4 | 90.7 | 8.3 | 90.5 | 8.3 |  |  |
| Prevalence | 0.7 | 0.2 | 0.7 | 0.2 | 0.7 | 0.2 | 0.7 | 0.2 | 0.8 | 0.3 | 0.8 | 0.3 |  |  |
| **Stroke** | | | | | | | | | | | | | | |
| Blood pressure measured (stroke) | 93.8 | 4.8 | 94.3 | 4.5 | 94.7 | 3.8 | 95.0 | 3.8 | 95.0 | 3.9 | 94.6 | 3.9 |  |  |
| Blood pressure controlled (stroke) | 82.3 | 7.7 | 83.6 | 6.8 | 85.0 | 6.5 | 85.9 | 6.3 | 86.1 | 6.2 | 85.7 | 6.2 | 85.2 | 5.8 |
| Cholesterol measured (stroke) | 86.3 | 8.0 | 87.0 | 7.3 | 88.5 | 6.4 | 89.0 | 6.2 | 88.7 | 6.4 | 88.2 | 6.4 | 88.0 | 6.4 |
| Cholesterol controlled (stroke) | 65.6 | 10.5 | 68.5 | 9.0 | 70.6 | 8.4 | 70.7 | 8.1 | 71.2 | 7.9 | 70.0 | 7.9 | 69.9 | 7.8 |
| Antiplatelet therapy (stroke) | 90.7 | 5.7 | 90.7 | 5.9 | 91.6 | 5.1 | 92.5 | 4.4 | 92.4 | 4.2 | 91.8 | 4.2 | 91.7 | 4.2 |
| Influenza immunisation (Stroke) | 75.6 | 8.7 | 73.9 | 9.0 | 74.9 | 8.5 | 76.5 | 7.9 | 77.4 | 7.4 | 77.6 | 7.4 | 78.2 | 7.2 |
| Prevalence | 1.9 | 0.6 | 2.0 | 0.6 | 2.0 | 0.6 | 2.1 | 0.6 | 2.1 | 0.6 | 2.2 | 0.6 | 2.2 | 0.6 |
