# Supplementary material for: The impact of quality and accessibility of primary care on emergency admissions for a range of chronic ambulatory care sensitive conditions (ACSCs) in Scotland: longitudinal analysis
Source: BMC Fam Pract. 2019 Feb 22;20:32. doi: 10.1186/s12875-019-0921-z (PMC6385424; doi:10.1186/s12875-019-0921-z)
Supplement: Supplementary file 5 — Full regression results. Full regression results including the covariates. (DOCX 34 kb) [file 12875_2019_921_MOESM5_ESM.docx]

**Additional file 5. Full regression results**

|  | **Asthma** | **COPD** | **Diabetes complication** | **Convulsions and Epilepsy** | **Hypertension** | **Stroke** | **Angina** |
| --- | --- | --- | --- | --- | --- | --- | --- |
| **Quality of clinical care** |  |  |  |  |  |  |  |
| Asthma review | -0.00189 |  |  |  |  |  |  |
| FEV 1 measurement (COPD) |  | -0.00102 |  |  |  |  |  |
| Inhaler technique check (COPD) |  | -2.00e-05 |  |  |  |  |  |
| Influenza immunisation (COPD) |  | -0.000256 |  |  |  |  |  |
| HbA1c measured (DM) |  |  | 0.00666 |  |  |  |  |
| HbA1c≤7/7.5 (DM) |  |  | 0.00196 |  |  |  |  |
| 7/7.5<HbA1c≤9/10 (DM) |  |  | -0.0112** |  |  |  |  |
| Blood pressure measured (DM) |  |  | 0.00950 |  |  |  |  |
| Blood pressure controlled (DM) |  |  | 0.00281 |  |  |  |  |
| Total cholesterol measured (DM) |  |  | -0.0118 |  |  |  |  |
| Total cholesterol controlled (DM) |  |  | -0.00650** |  |  |  |  |
| Influenza immunisation (DM) |  |  | 0.00242 |  |  |  |  |
| Epilepsy medication review |  |  |  | -0.00287** |  |  |  |
| Blood pressure measured (Hypertension) |  |  |  |  | -0.00431 |  |  |
| Blood pressure controlled (Hypertension) |  |  |  |  | -0.000582 |  |  |
| Blood pressure measured (Stroke) |  |  |  |  |  | 0.000760 |  |
| Blood pressure controlled (Stroke) |  |  |  |  |  | -0.000414 |  |
| Total cholesterol measured (Stroke) |  |  |  |  |  | 0.000919 |  |
| Total cholesterol controlled (Stroke) |  |  |  |  |  | -0.000587 |  |
| Antiplatelet therapy (Stroke) |  |  |  |  |  | 0.00440* |  |
| Influenza immunisation (Stroke) |  |  |  |  |  | -0.00460*** |  |
| Assessment for angina (CHD) |  |  |  |  |  |  | 0.000431 |
| Blood pressure measured (CHD) |  |  |  |  |  |  | -0.00223 |
| Blood pressure controlled (CHD) |  |  |  |  |  |  | -0.00196 |
| Total cholesterol measured (CHD) |  |  |  |  |  |  | 0.00526 |
| Total cholesterol controlled (CHD) |  |  |  |  |  |  | -0.00432* |
| Antiplatelet therapy (CHD) |  |  |  |  |  |  | 0.00748* |
| Betablocker therapy CHD) |  |  |  |  |  |  | 0.00464** |
| ACE inhibitor therapy (CHD) |  |  |  |  |  |  | 0.000296 |
| Influenza immunisation (CHD) |  |  |  |  |  |  | -0.00479 |
| **Access to primary care** |  |  |  |  |  |  |  |
| Weighted Drive time to nearest GP practice | -0.000348 | -0.00200 | 0.00128 | 0.00941** | -0.0253* | 0.00251 | 0.00680* |
| 48 hour GP access | -0.00555** | -0.00261 | 0.00147 | -0.000648 | -0.00957** | 3.61e-05 | -0.00220 |
| Advance Appointment | -0.00128 | 7.23e-05 | -0.000115 | 0.000559 | -0.00472** | -0.000606 | -0.00216*** |
| Continuity of care | -0.000540 | -0.000801 | -0.00236 | -0.00256 | -0.00346 | -0.00111 | 0.000764 |
| **Covariates** |  |  |  |  |  |  |  |
| Weighted distance to nearest 5 hospitals | -0.00760* | -0.00936** | -0.00143 | -0.00861*** | 0.00700 | -0.00590** | -0.00407 |
| ER Asthma review | -0.00175 |  |  |  |  |  |  |
| ER FEV 1 measurement (COPD) |  | -4.36e-05 |  |  |  |  |  |
| ER Inhaler technique check (COPD) |  | -0.000349 |  |  |  |  |  |
| ER Influenza immunisation (COPD) |  | 0.00133 |  |  |  |  |  |
| ER HbA1c measured (DM) |  |  | -0.00208 |  |  |  |  |
| ER HbA1c≤7/7.5 (DM) |  |  | 9.14e-05 |  |  |  |  |
| ER 7/7.5<HbA1c≤9/10 (DM) |  |  | -0.000891 |  |  |  |  |
| ER Blood pressure measured (DM) |  |  | 0.0335** |  |  |  |  |
| ER Blood pressure controlled (DM) |  |  | -0.000977 |  |  |  |  |
| ER Total cholesterol measured (DM) |  |  | -0.00719 |  |  |  |  |
| ER Total cholesterol controlled (DM) |  |  | 0.000514 |  |  |  |  |
| ER Influenza immunisation (DM) |  |  | -0.000644 |  |  |  |  |
| ER Assessment for angina (CHD) |  |  |  |  |  |  | 0.000807 |
| ER Blood pressure measured (CHD) |  |  |  |  |  |  | 0.00189 |
| ER Blood pressure controlled (CHD) |  |  |  |  |  |  | -0.0106 |
| ER Total cholesterol measured (CHD) |  |  |  |  |  |  | 0.00354 |
| ER Total cholesterol controlled (CHD) |  |  |  |  |  |  | 0.00190 |
| ER Antiplatelet therapy (CHD) |  |  |  |  |  |  | -0.00629 |
| ER Betablocker therapy CHD) |  |  |  |  |  |  | 0.00175 |
| ER ACE inhibitor therapy (CHD) |  |  |  |  |  |  | 9.59e-05 |
| ER Influenza immunisation (CHD) |  |  |  |  |  |  | -0.00295 |
| ER Epilepsy medication review |  |  |  | 0.000132 |  |  |  |
| ER Blood pressure measured (Hypertension) |  |  |  |  | 0.00822 |  |  |
| ER Blood pressure controlled (Hypertension) |  |  |  |  | -0.00663 |  |  |
| ER Blood pressure measured (Stroke) |  |  |  |  |  | -0.00149 |  |
| ER Blood pressure controlled (Stroke) |  |  |  |  |  | 0.000422 |  |
| ER Total cholesterol measured (Stroke) |  |  |  |  |  | 0.000375 |  |
| ER Total cholesterol controlled (Stroke) |  |  |  |  |  | 0.00198 |  |
| ER Antiplatelet therapy (Stroke) |  |  |  |  |  | 0.00302 |  |
| ER Influenza immunisation (Stroke) |  |  |  |  |  | -0.00551*** |  |
| Prevalence rate | 0.0332*** | 0.113*** | 0.109*** | 0.409*** | -0.00502 | 0.0979*** | 0.118*** |
| Pre QOF admission rate | 0.0262*** | 0.00936*** | 0.0244*** | 0.0116*** | 0.0640*** | 0.00678*** | 0.0108*** |
| Practice population | -5.79e-07 | 5.27e-06 | -7.28e-06 | -3.43e-06 | -1.81e-05** | -8.63e-06** | -7.58e-06* |
| Practice population per GP | -5.34e-05 | -2.38e-06 | -3.54e-05 | 1.71e-05 | -5.57e-05 | 2.36e-05 | -3.74e-05 |
| GMS practice | -0.00720 | 0.0221 | 0.0112 | 0.0150 | -0.117 | -0.00339 | -0.0681** |
| Average physician age | 0.000419 | 0.000541 | -0.00556* | -0.00218 | -0.00162 | 0.00164 | -0.00272 |
| Proportion of female physicians | -0.0438 | 0.0221 | -0.191*** | -0.0399 | 0.0707 | 0.0117 | -0.0171 |
| Proportion of principal physicians | -0.102 | -0.100* | 0.0905 | 0.0240 | -0.0690 | 0.0141 | -0.00705 |
| Dispensing GP | 0.0124 | -0.0589 | 0.0344 | 0.00298 | 0.00148 | 0.0582 | -0.0502 |
| Male 0-4(%) | 0.0292 | -4.97e-05 | 0.0226 | 0.0872*** | -0.0761 | 0.00701 | -0.0665** |
| Male 5-14(%) | -0.0117 | 0.0406* | 0.0105 | -0.00607 | -0.0261 | 0.0208 | 0.0354 |
| Male 25-44(%) | 0.0206** | -0.00926 | 0.0149 | -0.000845 | -0.00408 | -0.000862 | 0.00786 |
| Male 45-64(%) | -0.00893 | 0.0241** | 0.0142 | 0.0350*** | 0.0642*** | 0.0217*** | 0.00166 |
| Male 65-74(%) | 0.0782*** | 0.0124 | -0.0349 | -0.0311 | -0.0665 | 0.0473*** | 0.0216 |
| Male 75-84(%) | 0.0328 | 0.0479 | 0.0379 | 0.0484 | 0.00593 | 0.0387 | -0.00849 |
| Male 85+(%) | -0.0496 | -0.0224 | 0.0877 | 0.212*** | 0.217 | -0.00138 | 0.0404 |
| Female 0-4(%) | 0.0799** | -0.0339 | 0.0640 | -0.0263 | 0.0251 | 0.0353 | 0.0459 |
| Female 5-14(%) | 0.0675** | -0.0153 | -0.0172 | 0.0258 | 0.0832 | -0.0153 | 0.0338 |
| Female 25-44(%) | 0.00313 | 0.00476 | -0.0144 | 0.0217** | 0.000702 | 0.00399 | -0.00587 |
| Female 45-64(%) | 0.0610*** | -0.0158 | 0.0424** | 0.000127 | -0.0433* | 0.0160 | 0.0392*** |
| Female 65-74(%) | -0.0758*** | -0.0219 | -0.0220 | 0.0220 | 0.112* | 0.0443*** | -0.0584** |
| Female 75-84(%) | 0.0362 | 0.0586** | 0.00763 | 0.00249 | 0.0204 | 0.0402** | 0.0503* |
| Female 85(%) | 0.000956 | 0.0169 | -0.0296 | -0.0518 | -0.0422 | 0.0575** | 0.0397 |
| Income deprivation | 0.0136 | -0.0100 | -0.0274 | 0.00274 | -0.00984 | 0.00902 | 0.000378 |
| Education deprivation | 0.135* | 0.341*** | 0.201** | 0.218*** | 0.0453 | 0.0517 | 0.170*** |
| Housing deprivation | 0.00203 | 0.000575 | -0.00310 | -0.00661* | 0.00352 | 0.00273 | 0.000670 |
| Crime | -4.25e-05 | -4.32e-05 | -0.000100** | -4.77e-05 | -2.78e-05 | -3.26e-06 | -8.71e-05*** |
| Employment deprivation | -0.00937 | 0.0236 | 0.0342 | 0.0106 | 0.0159 | -0.000947 | -0.00890 |
| Other Urban Areas | -0.00354 | 0.0837 | -0.0645 | 0.00625 | 0.0774 | -0.00484 | 0.0346 |
| Accessible Small Towns | -0.210** | 0.125* | -0.189** | -0.111 | 0.0610 | -0.0192 | -0.0312 |
| Remote Small Towns | -0.0371 | -0.0244 | -0.205* | -0.311*** | 0.563*** | 0.0356 | 0.0340 |
| Accessible Rural | -0.127 | 0.124 | -0.162 | -0.179* | -0.104 | -0.109* | -0.0254 |
| Remote Rural | -0.266** | 0.0923 | -0.265* | -0.286*** | 0.284 | -0.215*** | -0.202* |
| Year: 2006/07 | 0.193*** | 0.0950*** | 0.0173 | 0.0197 | -0.0216 | -0.0307 | -0.0415 |
| Year: 2007/08 | 0.0274 | 0.102*** | -0.00872 | 0.0154 | -0.159** | -0.0727*** | 0.137*** |
| Year: 2008/09 | 0.102*** | 0.184*** | -0.0335 | 0.0411 | -0.131* | -0.0457** | 0.0545* |
| Year: 2009/10 | -0.0340 | 0.111*** | -0.138*** | -0.0516 | -0.0677 | -0.0547** | -0.141*** |
| Year: 2010/11 | -0.0644* | 0.138*** | -0.102* | -0.156*** | -0.166* | -0.0663** | -0.269*** |
| Year: 2011/12 | -0.137*** | 0.171*** |  |  | -0.176* |  |  |
| Constant | -8.233*** | -6.320*** | -7.333*** | -7.022*** | -8.230*** | -8.460*** | -7.821*** |
| Observations | 6,084 | 6,050 | 5,236 | 5,216 | 6,083 | 5,243 | 4,908 |
| Number of practices | 907 | 903 | 906 | 904 | 907 | 906 | 888 |

Coefficients from GEE negative binomial regression models. The coefficients on the quality indicators and access measures show the proportionate change in % admissions associated with a 1% increase in population achievement. *** p<0.01, ** p<0.05, * p<0.1
