# Supplementary material for: The impact of quality and accessibility of primary care on emergency admissions for a range of chronic ambulatory care sensitive conditions (ACSCs) in Scotland: longitudinal analysis
Source: BMC Fam Pract. 2019 Feb 22;20:32. doi: 10.1186/s12875-019-0921-z (PMC6385424; doi:10.1186/s12875-019-0921-z)
Supplement: Supplementary file 6 — Robustness checks - sign and significance level of coefficients. Robustness checks of the results. (DOCX 27 kb) [file 12875_2019_921_MOESM6_ESM.docx]

**Additional file 6. Robustness checks - sign and significance level of coefficients**

|  | **Pooled cross sectional negative binomial** | **Fixed effects linear regression – admission rate** | **Random effects linear regression – admission rate** |
| --- | --- | --- | --- |
| **Asthma** |  |  |  |
| Patient reviews | -; 10% | -; NS | -; 10% |
| Drive time to nearest GP practice | +; NS | -; 1% | -; NS |
| Distance to nearest 5 hospitals | -; 1% | +; NS | -; NS |
| 48 hour GP access | -; 5% |  | -; 10% |
| Advance appointment | -; NS |  | -; NS |
| Continuity of Care | -; NS |  | -; NS |
| **COPD** |  |  |  |
| FEV 1 measurement | -; 5% | -; NS | -; NS |
| Inhaler technique | -; NS | +; NS | +; NS |
| Influenza immunisation (primary) | +; NS | -; NS | -; NS |
| Drive time to nearest GP practice | -; NS | -; NS | -; NS |
| Distance to nearest 5 hospitals | -; 5% | -; NS | -; 10% |
| 48 hour GP access | -; NS |  | -; NS |
| Advance appointment | -; NS |  | -; NS |
| Continuity of Care | +; NS |  | +; NS |
| **Diabetes complications** |  |  |  |
| HbA1c monitored | +; NS | +; NS | +; NS |
| HbA1c ≤ 7/7.5 | +; NS | +; NS | +; NS |
| 7/7.5 < HbA1c ≤ 9/10 | -; 1% | -; NS | -; 5% |
| Blood pressure measured (primary) | +; NS | +; NS | +; NS |
| Blood pressure controlled  (primary) | +; NS | +; 5% | +; 5% |
| Total cholesterol measured  (primary) | -; NS | -; NS | -; NS |
| Total cholesterol controlled  (primary) | -; NS | -; 5% | -; 5% |
| Influenza immunisation (primary) | +; NS | +; NS | -; NS |
| Specialist assessment for newly diagnosed angina | -; 10% | -; NS | -; NS |
| Drive time to nearest GP practice | +; NS | -; NS | -; NS |
| Distance to nearest 5 hospitals | -; NS | +; NS | -; NS |
| 48 hour GP access | +; NS |  | +; NS |
| Advance appointment | -; NS |  | -; NS |
| Continuity of Care | -; NS |  | -; NS |
| **Convulsions & epilepsy** |  |  |  |
| Medication review | -; NS | -; 5% | -; NS |
| Drive time to nearest GP practice | +; 5% | -; 10% | +; NS |
| Distance to nearest 5 hospitals | -; 1% | -; NS | -; 5% |
| 48 hour GP access | -; NS | -; NS | -; NS |
| Advance appointment | +; NS | +; NS | +; NS |
| Continuity of Care | -; 10% | -; NS | -; NS |
| **Hypertension** |  |  |  |
| Blood pressure measured (primary) | -; NS | +; NS | +; NS |
| Blood pressure controlled (primary) | -; NS | -; NS | -; NS |
| Drive time to nearest GP practice | -; NS | -; NS | -; 5% |
| Distance to nearest 5 hospitals | +; NS | +; NS | +; 5% |
| 48 hour GP access | -; 5% |  | -; 5% |
| Advance appointment | -; 5% |  | -; NS |
| Continuity of Care | -; NS |  | -; NS |
| **Stroke** |  |  |  |
| Blood pressure measured (primary) | -; NS | +; 10% | +; NS |
| Blood pressure controlled (primary) | +; NS | -; NS | -; NS |
| Total cholesterol measured | -; NS | -; NS | +; NS |
| Total cholesterol controlled | -; NS | +; NS | -; NS |
| Antiplatelet therapy | +; 1% | +; 10% | +; 5% |
| Influenza immunisation (primary) | -; 10% | -; 1% | -; 1% |
| Drive time to nearest GP practice | -; NS | -; NS | +; NS |
| Distance to nearest 5 hospitals | -; 10% | -; NS | -; 1% |
| 48 hour GP access | +; NS |  | -; NS |
| Advance appointment | -; NS |  | -; NS |
| Continuity of Care | -; NS |  | -; NS |
| **Angina** |  |  |  |
| Specialist assessment for newly diagnosed angina | +; NS | -; NS | +; NS |
| Blood pressure measured (primary) | -; NS | -; NS | -; NS |
| Blood pressure controlled (primary) | +; NS | +; NS | -; NS |
| Total cholesterol measured (primary) | +; NS | +; NS | +; NS |
| Total cholesterol controlled (primary) | -; 10% | -; NS | -; 5% |
| Antiplatelet therapy | +; 5% | +; 1% | +; 5% |
| Beta blocker therapy | +; NS | +; 5% | +; 5% |
| ACE inhibitor therapy | +; NS | +; NS | +; NS |
| Influenza immunisation (primary) | -; NS | -; 5% | -; 5% |
| Drive time to nearest GP practice | +; NS | +; NS | +; NS |
| Distance to nearest 5 hospitals | -; NS | -; NS | -; NS |
| 48 hour GP access | -; NS |  | -; NS |
| Advance appointment | -; 1% |  | -; NS |
| Continuity of Care | +; NS |  | +; NS |
| Sign of coefficient; Significance level (NS is Not Significant). | | | |
